# Bis(μ-imino­diacetato)bis­[(2,2′-diamino-4,4′-bi-1,3-thia­zole)lead(II)] tetra­hydrate

**DOI:** 10.1107/S1600536810006926

**Published:** 2010-02-27

**Authors:** Mei Du, Bing-Xin Liu, Jing-Jing Nie, Duan-Jun Xu

**Affiliations:** a Department of Chemistry, Shanghai University, People’s Republic of China; b Department of Chemistry, Zhejiang University, People’s Republic of China

## Abstract

In the crystal structure of the title compound, [Pb_2_(C_4_H_5_NO_4_)_2_(C_6_H_6_N_4_S_2_)_2_]·4H_2_O, the dinuclear Pb^II^ complex mol­ecule is centrosymmetric. The Pb atom is chelated by a tridentate imino­diacetate anion (IDA) and a diamino­bithia­zole (DABT) ligand, while a carboxyl­ate O atom from an adjacent IDA anion further bridges the Pb atom with a longer Pb—O bond [2.892 (3) Å]. The lone-pair electrons of the Pb atom occupy an axial site in the Ψ-penta­gonal-bipyramidal coordination polyhedron. The IDA anion displays a facial configuration: its chelating five-membered rings assume an envelope configuration. Within the DABT ligand, the two thia­zole rings are twisted relative to each other, making a dihedral angle of 9.51 (17)°. Extensive N—H⋯O, O—H⋯O and weak C—H⋯O hydrogen bonding helps to stabilize the crystal structure.

## Related literature

For the potential applications of metal complexes of diamino­bithia­zole in the field of biology, see: Waring (1981 ▶); Fisher *et al.* (1985 ▶). For Pb^II^ complexes with a similar coordination geometry, see: Lacouture *et al.* (2001 ▶); Jones *et al.* (1988 ▶). For a complex with a longer Pb—O bond distance [2.968 (4) Å], see: Inoue *et al.* (1993 ▶). For the dihedral angles between thia­zole rings in diamino­bithia­zole complexes, see: Liu *et al.* (2006 ▶); Zhang *et al.* (2006 ▶).
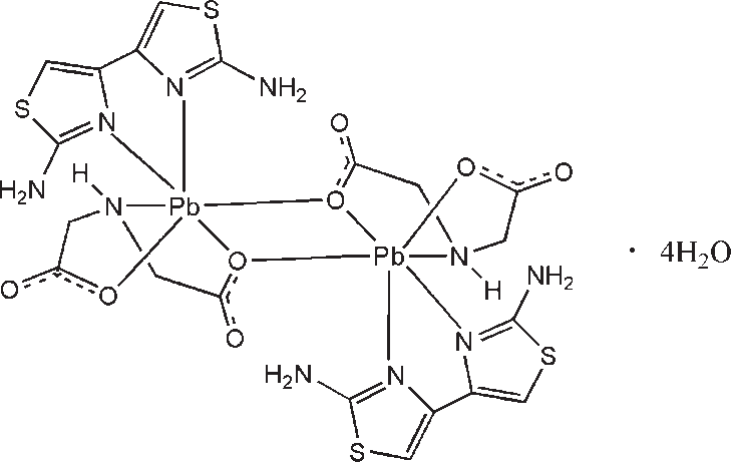


## Experimental

### 

#### Crystal data

[Pb_2_(C_4_H_5_NO_4_)_2_(C_6_H_6_N_4_S_2_)_2_]·4H_2_O
*M* *_r_* = 1145.16
Triclinic, 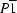

*a* = 9.2241 (8) Å
*b* = 9.8526 (9) Å
*c* = 10.6380 (11) Å
α = 77.0732 (12)°
β = 67.4141 (15)°
γ = 69.0690 (12)°
*V* = 829.54 (14) Å^3^
*Z* = 1
Mo *K*α radiation
μ = 10.46 mm^−1^
*T* = 295 K
0.20 × 0.12 × 0.10 mm

#### Data collection

Rigaku R-AXIS RAPID diffractometer
Absorption correction: multi-scan (*ABSCOR*; Higashi, 1995 ▶) *T*_min_ = 0.132, *T*_max_ = 0.350
5960 measured reflections
2878 independent reflections
2745 reflections with *I* > 2σ(*I*)
*R*_int_ = 0.021

#### Refinement

*R*[*F* ^2^ > 2σ(*F* ^2^)] = 0.020
*wR*(*F* ^2^) = 0.048
*S* = 1.07
2878 reflections
244 parameters
9 restraints
H atoms treated by a mixture of independent and constrained refinement
Δρ_max_ = 0.54 e Å^−3^
Δρ_min_ = −0.70 e Å^−3^

### 

Data collection: *PROCESS-AUTO* (Rigaku, 1998 ▶); cell refinement: *PROCESS-AUTO*; data reduction: *CrystalStructure* (Rigaku/MSC, 2002 ▶); program(s) used to solve structure: *SIR92* (Altomare *et al.*, 1993 ▶); program(s) used to refine structure: *SHELXL97* (Sheldrick, 2008 ▶); molecular graphics: *ORTEP-3 for Windows* (Farrugia, 1997 ▶); software used to prepare material for publication: *WinGX* (Farrugia, 1999 ▶).

## Supplementary Material

Crystal structure: contains datablocks I, global. DOI: 10.1107/S1600536810006926/ng2733sup1.cif

Structure factors: contains datablocks I. DOI: 10.1107/S1600536810006926/ng2733Isup2.hkl

Additional supplementary materials:  crystallographic information; 3D view; checkCIF report

## Figures and Tables

**Table 1:** Selected bond lengths (Å)

Pb—O1	2.546 (3)
Pb—O1^i^	2.892 (3)
Pb—O3	2.536 (3)
Pb—N1	2.593 (3)
Pb—N3	2.594 (3)
Pb—N5	2.402 (4)

Symmetry code: (i) 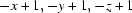.

**Table 2:** Hydrogen-bond geometry (Å, °)

*D*—H⋯*A*	*D*—H	H⋯*A*	*D*⋯*A*	*D*—H⋯*A*
N2—H2*A*⋯O3	0.86 (6)	2.05 (5)	2.880 (6)	162 (5)
N2—H2*B*⋯O1*W*	0.86 (3)	2.16 (4)	2.959 (6)	153 (6)
N4—H4*A*⋯O1	0.86 (5)	2.15 (5)	2.946 (5)	155 (5)
N4—H4*B*⋯O4^ii^	0.86 (3)	2.07 (5)	2.885 (7)	159 (6)
N5—H5*N*⋯O2*W*	0.87 (5)	2.01 (6)	2.809 (7)	153 (4)
O1*W*—H11⋯O2^iii^	0.82 (2)	1.97 (2)	2.783 (6)	171 (5)
O1*W*—H12⋯O3^iv^	0.82 (5)	2.11 (4)	2.819 (6)	144 (5)
O2*W*—H21⋯O1*W*^v^	0.82 (7)	2.10 (7)	2.892 (7)	161 (6)
O2*W*—H22⋯O4^vi^	0.82 (5)	1.95 (5)	2.766 (6)	168 (7)
C5—H5⋯O2^vii^	0.93	2.56	3.476 (6)	167

Symmetry codes: (ii) 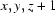; (iii) 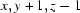; (iv) 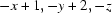; (v) 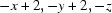; (vi) 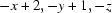; (vii) 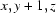.

**Fig. 1.**
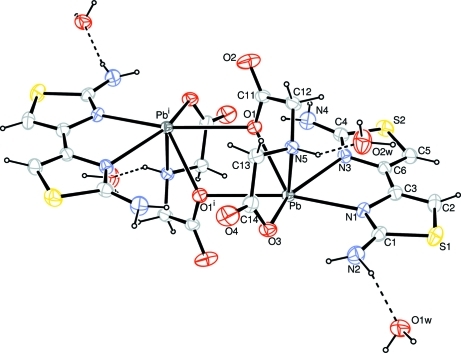
The dinuclear molecular structure with 30% probability displacement ellipsoids. Dashed lines indicate the hydrogen bonding [symmetric code: (i) -x,-y,1-z].

## supplementary crystallographic information

### Comment

Some metal complexes with 2,2'-diamino-4,4'-bi-1,3-thiazole (DABT) have shown the potential application in the biological field (Waring, 1981; Fisher *et al.*, 1985). As a part of serial structural investigation of metal complexes with DABT, the title Pb^II^ complex was prepared in the laboratory and its X-ray structure is presented here.

The molecular structure of the title compound is shown in Fig. 1. The dinuclear Pb^II^ complex molecule is centro-symmetric. Each Pb atom is chelated by a tridentate iminodiacetate anion (IDA) and a diaminobithiazole (DABT) ligand, and one carboxyl O atom from the adjacent IDA anion further bridges the Pb atom. The lone-pair electrons of the Pb atom occupy an axial site in the distorted Ψ-pentagonal bipyramidal coordination geometry, which is similar to that found in Pb^II^ complexes reported previously (Lacouture *et al.*, 2001; Jones *et al.*, 1988). The longer Pb—O(bridge) bond distance (Table 1) is comparable to 2.968 (4) Å found in a related Pb complex (Inoue *et al.*, 1993). The IDA displays a facial configuration, its both chelating five-membered rings assume the envelope configuration. Within the DABT ligand, the two thiazole rings are twisted to each other with a dihedral angle of 9.51 (17)°, it agrees with 14.7 (3) and 9.5 (2)° found in transition metal complexes of DABT (Liu *et al.*, 2006; Zhang *et al.*, 2006).

The extensive N—H···O, O—H···O and weak C—H···O hydrogen bonding helps to stabilize the crystal structure (Table 2).

### Experimental

An aqueous solution (20 ml) containing DABT (0.20 g, 1 mmol) and Pb(NO_3_)_2_ (0.33 g, 1 mmol) was mixed with another aqueous solution (10 ml) of H_2_IDA (0.13 g, 1 mmol) and NaOH (0.08 g, 2 mmol). The mixture was refluxed for 5 h. The solution was filtered after cooling to room temperature. Single crystals were obtained from the filtrate after one week.

### Refinement

H atoms bonded to N and O atoms were located in a difference Fourier map and were refined with distance constraints [O—H = 0.82±0.03 and N—H = 0.86±0.03 Å] and U_iso_(H) = 0.08 Å^2^. H atoms on carbon atoms were placed in calculated positions with C—H = 0.97 Å (methylene) and 0.93 Å (aromatic), and included in the final cycles of refinement in the riding model with U_iso_(H) = 1.2U_eq_(C).

### Crystal data

**Table:**

[Pb_2_(C_4_H_5_NO_4_)_2_(C_6_H_6_N_4_S_2_)_2_]·4H_2_O	*Z* = 1
*M**_r_* = 1145.16	*F*(000) = 544
Triclinic, *P*1	*D*_x_ = 2.292 Mg m^−^^3^
Hall symbol: -P 1	Mo *K*α radiation, λ = 0.71073 Å
*a* = 9.2241 (8) Å	Cell parameters from 4336 reflections
*b* = 9.8526 (9) Å	θ = 2.1–24.6°
*c* = 10.6380 (11) Å	µ = 10.46 mm^−^^1^
α = 77.0732 (12)°	*T* = 295 K
β = 67.4141 (15)°	Block, yellow
γ = 69.0690 (12)°	0.20 × 0.12 × 0.10 mm
*V* = 829.54 (14) Å^3^	

### Data collection

**Table:**

Rigaku R-AXIS RAPID diffractometer	2878 independent reflections
Radiation source: fine-focus sealed tube	2745 reflections with *I* > 2σ(*I*)
graphite	*R*_int_ = 0.021
Detector resolution: 10.00 pixels mm^-1^	θ_max_ = 25.0°, θ_min_ = 2.1°
ω scans	*h* = −10→10
Absorption correction: multi-scan (*ABSCOR*; Higashi, 1995)	*k* = −11→11
*T*_min_ = 0.132, *T*_max_ = 0.350	*l* = −12→12
5960 measured reflections	

### Refinement

**Table:**

Refinement on *F*^2^	Primary atom site location: structure-invariant direct methods
Least-squares matrix: full	Secondary atom site location: difference Fourier map
*R*[*F*^2^ > 2σ(*F*^2^)] = 0.020	Hydrogen site location: inferred from neighbouring sites
*wR*(*F*^2^) = 0.048	H atoms treated by a mixture of independent and constrained refinement
*S* = 1.07	*w* = 1/[σ^2^(*F*_o_^2^) + (0.019*P*)^2^ + 0.7917*P*] where *P* = (*F*_o_^2^ + 2*F*_c_^2^)/3
2878 reflections	(Δ/σ)_max_ = 0.001
244 parameters	Δρ_max_ = 0.54 e Å^−^^3^
9 restraints	Δρ_min_ = −0.70 e Å^−^^3^

### Special details

**Table:**

Geometry. All esds (except the esd in the dihedral angle between two l.s. planes) are estimated using the full covariance matrix. The cell esds are taken into account individually in the estimation of esds in distances, angles and torsion angles; correlations between esds in cell parameters are only used when they are defined by crystal symmetry. An approximate (isotropic) treatment of cell esds is used for estimating esds involving l.s. planes.
Refinement. Refinement of *F*^2^ against ALL reflections. The weighted *R*-factor wR and goodness of fit *S* are based on *F*^2^, conventional *R*-factors *R* are based on *F*, with *F* set to zero for negative *F*^2^. The threshold expression of *F*^2^ > σ(*F*^2^) is used only for calculating *R*-factors(gt) etc. and is not relevant to the choice of reflections for refinement. *R*-factors based on *F*^2^ are statistically about twice as large as those based on *F*, and *R*- factors based on ALL data will be even larger.

### Fractional atomic coordinates and isotropic or equivalent isotropic displacement parameters (Å^2^)

**Table:**

	*x*	*y*	*z*	*U*_iso_*/*U*_eq_	
Pb	0.545731 (18)	0.724630 (15)	0.424338 (14)	0.02919 (7)	
S1	0.85653 (18)	1.10157 (14)	0.16319 (14)	0.0551 (3)	
S2	0.70556 (17)	0.84111 (15)	0.78711 (13)	0.0513 (3)	
O1	0.6288 (4)	0.4760 (3)	0.5554 (3)	0.0400 (7)	
O2	0.8401 (5)	0.2774 (4)	0.5652 (4)	0.0696 (11)	
O3	0.5809 (4)	0.7154 (3)	0.1777 (3)	0.0431 (7)	
O4	0.6770 (6)	0.5550 (4)	0.0268 (4)	0.0714 (12)	
O1W	0.7380 (5)	1.1758 (4)	−0.1588 (4)	0.0577 (9)	
O2W	1.0741 (5)	0.6671 (5)	0.1186 (5)	0.0717 (11)	
N1	0.7119 (4)	0.9098 (4)	0.3108 (4)	0.0359 (8)	
N2	0.7160 (6)	0.9566 (5)	0.0832 (4)	0.0541 (11)	
N3	0.6755 (4)	0.7770 (4)	0.5787 (3)	0.0357 (8)	
N4	0.5919 (6)	0.6270 (5)	0.7807 (4)	0.0563 (11)	
N5	0.8084 (4)	0.5688 (4)	0.2998 (4)	0.0355 (8)	
C1	0.7507 (5)	0.9781 (5)	0.1862 (4)	0.0382 (10)	
C2	0.8435 (6)	1.0598 (5)	0.3335 (5)	0.0494 (12)	
H2	0.8841	1.1027	0.3770	0.059*	
C3	0.7666 (5)	0.9569 (5)	0.3947 (4)	0.0372 (10)	
C4	0.6525 (6)	0.7362 (5)	0.7099 (4)	0.0399 (10)	
C5	0.7564 (6)	0.9461 (5)	0.6339 (5)	0.0461 (11)	
H5	0.7934	1.0262	0.6206	0.055*	
C6	0.7354 (5)	0.8967 (4)	0.5350 (5)	0.0368 (10)	
C11	0.7810 (6)	0.4004 (5)	0.5131 (4)	0.0370 (10)	
C12	0.8948 (6)	0.4667 (5)	0.3912 (5)	0.0418 (10)	
H12A	0.9443	0.5183	0.4229	0.050*	
H12B	0.9827	0.3892	0.3400	0.050*	
C13	0.7855 (6)	0.4939 (5)	0.2072 (4)	0.0408 (10)	
H13A	0.7393	0.4162	0.2599	0.049*	
H13B	0.8919	0.4499	0.1420	0.049*	
C14	0.6733 (6)	0.5959 (5)	0.1303 (4)	0.0427 (11)	
H11	0.768 (8)	1.197 (7)	−0.2418 (10)	0.080*	
H12	0.6393 (18)	1.220 (6)	−0.132 (6)	0.080*	
H21	1.124 (7)	0.724 (5)	0.113 (7)	0.080*	
H22	1.147 (6)	0.594 (4)	0.086 (7)	0.080*	
H2A	0.662 (6)	0.895 (5)	0.101 (6)	0.080*	
H2B	0.751 (8)	0.997 (6)	0.001 (2)	0.080*	
H4A	0.582 (7)	0.570 (5)	0.736 (5)	0.080*	
H4B	0.595 (7)	0.598 (6)	0.862 (2)	0.080*	
H5N	0.865 (6)	0.626 (5)	0.247 (5)	0.080*	

### Atomic displacement parameters (Å^2^)

**Table:**

	*U*^11^	*U*^22^	*U*^33^	*U*^12^	*U*^13^	*U*^23^
Pb	0.03072 (10)	0.03010 (10)	0.02682 (10)	−0.00792 (7)	−0.01080 (7)	−0.00292 (6)
S1	0.0619 (9)	0.0475 (7)	0.0531 (7)	−0.0305 (6)	−0.0092 (6)	0.0054 (6)
S2	0.0594 (8)	0.0592 (8)	0.0430 (7)	−0.0105 (6)	−0.0248 (6)	−0.0183 (6)
O1	0.0430 (19)	0.0399 (17)	0.0384 (17)	−0.0150 (15)	−0.0159 (14)	0.0016 (13)
O2	0.070 (3)	0.051 (2)	0.064 (2)	−0.0008 (19)	−0.025 (2)	0.0136 (19)
O3	0.051 (2)	0.0448 (18)	0.0352 (16)	−0.0102 (15)	−0.0218 (15)	−0.0005 (14)
O4	0.110 (3)	0.068 (2)	0.049 (2)	−0.016 (2)	−0.046 (2)	−0.0134 (19)
O1W	0.063 (2)	0.059 (2)	0.054 (2)	−0.0169 (19)	−0.030 (2)	0.0050 (19)
O2W	0.062 (3)	0.076 (3)	0.074 (3)	−0.035 (2)	−0.002 (2)	−0.013 (2)
N1	0.040 (2)	0.0315 (18)	0.0362 (19)	−0.0136 (16)	−0.0107 (16)	−0.0017 (15)
N2	0.068 (3)	0.058 (3)	0.039 (2)	−0.030 (2)	−0.019 (2)	0.010 (2)
N3	0.038 (2)	0.041 (2)	0.0309 (18)	−0.0143 (16)	−0.0126 (16)	−0.0041 (15)
N4	0.081 (3)	0.066 (3)	0.032 (2)	−0.033 (3)	−0.026 (2)	0.007 (2)
N5	0.034 (2)	0.039 (2)	0.035 (2)	−0.0125 (16)	−0.0138 (16)	−0.0001 (16)
C1	0.038 (2)	0.033 (2)	0.037 (2)	−0.0107 (19)	−0.0072 (19)	−0.0009 (18)
C2	0.050 (3)	0.046 (3)	0.058 (3)	−0.024 (2)	−0.013 (2)	−0.010 (2)
C3	0.034 (2)	0.035 (2)	0.040 (2)	−0.0065 (19)	−0.0086 (19)	−0.0115 (19)
C4	0.043 (3)	0.047 (3)	0.032 (2)	−0.008 (2)	−0.015 (2)	−0.010 (2)
C5	0.052 (3)	0.044 (3)	0.051 (3)	−0.012 (2)	−0.024 (2)	−0.014 (2)
C6	0.033 (2)	0.034 (2)	0.045 (2)	−0.0053 (18)	−0.015 (2)	−0.0113 (19)
C11	0.043 (3)	0.038 (2)	0.034 (2)	−0.008 (2)	−0.021 (2)	−0.0024 (19)
C12	0.038 (3)	0.048 (3)	0.042 (2)	−0.012 (2)	−0.021 (2)	0.001 (2)
C13	0.049 (3)	0.040 (2)	0.036 (2)	−0.009 (2)	−0.019 (2)	−0.0080 (19)
C14	0.054 (3)	0.045 (3)	0.032 (2)	−0.020 (2)	−0.014 (2)	0.000 (2)

### Geometric parameters (Å, °)

**Table:**

Pb—O1	2.546 (3)	N2—H2A	0.86 (6)
Pb—O1^i^	2.892 (3)	N2—H2B	0.86 (3)
Pb—O3	2.536 (3)	N3—C4	1.318 (5)
Pb—N1	2.593 (3)	N3—C6	1.391 (5)
Pb—N3	2.594 (3)	N4—C4	1.335 (6)
Pb—N5	2.402 (4)	N4—H4A	0.86 (5)
S1—C2	1.732 (5)	N4—H4B	0.86 (3)
S1—C1	1.739 (4)	N5—C13	1.468 (5)
S2—C5	1.718 (5)	N5—C12	1.475 (5)
S2—C4	1.741 (4)	N5—H5N	0.86 (5)
O1—C11	1.283 (5)	C2—C3	1.348 (6)
O2—C11	1.237 (5)	C2—H2	0.9300
O3—C14	1.259 (5)	C3—C6	1.435 (6)
O4—C14	1.239 (5)	C5—C6	1.354 (6)
O1W—H11	0.819 (16)	C5—H5	0.9300
O1W—H12	0.82 (5)	C11—C12	1.509 (6)
O2W—H21	0.82 (7)	C12—H12A	0.9700
O2W—H22	0.82 (5)	C12—H12B	0.9700
N1—C1	1.321 (5)	C13—C14	1.513 (6)
N1—C3	1.403 (5)	C13—H13A	0.9700
N2—C1	1.331 (6)	C13—H13B	0.9700
			
N5—Pb—O3	66.12 (11)	N1—C1—S1	114.6 (3)
N5—Pb—O1	67.04 (11)	N2—C1—S1	120.7 (3)
O3—Pb—O1	114.56 (10)	C3—C2—S1	110.9 (4)
N5—Pb—N1	78.68 (11)	C3—C2—H2	124.5
O3—Pb—N1	81.95 (10)	S1—C2—H2	124.5
O1—Pb—N1	128.64 (10)	C2—C3—N1	115.0 (4)
N5—Pb—N3	90.68 (12)	C2—C3—C6	125.7 (4)
O3—Pb—N3	143.48 (11)	N1—C3—C6	119.3 (4)
O1—Pb—N3	77.56 (10)	N3—C4—N4	124.6 (4)
N1—Pb—N3	65.44 (11)	N3—C4—S2	113.7 (3)
C2—S1—C1	89.0 (2)	N4—C4—S2	121.7 (3)
C5—S2—C4	89.4 (2)	C6—C5—S2	111.0 (4)
C11—O1—Pb	116.9 (3)	C6—C5—H5	124.5
C14—O3—Pb	114.4 (3)	S2—C5—H5	124.5
H11—O1W—H12	105 (6)	C5—C6—N3	114.7 (4)
H21—O2W—H22	104 (6)	C5—C6—C3	126.4 (4)
C1—N1—C3	110.4 (4)	N3—C6—C3	119.0 (4)
C1—N1—Pb	132.4 (3)	O2—C11—O1	124.6 (4)
C3—N1—Pb	117.1 (3)	O2—C11—C12	118.1 (4)
C1—N2—H2A	116 (4)	O1—C11—C12	117.3 (4)
C1—N2—H2B	122 (4)	N5—C12—C11	112.3 (4)
H2A—N2—H2B	121 (6)	N5—C12—H12A	109.1
C4—N3—C6	111.2 (4)	C11—C12—H12A	109.1
C4—N3—Pb	128.8 (3)	N5—C12—H12B	109.1
C6—N3—Pb	116.8 (3)	C11—C12—H12B	109.1
C4—N4—H4A	118 (4)	H12A—C12—H12B	107.9
C4—N4—H4B	119 (4)	N5—C13—C14	112.6 (4)
H4A—N4—H4B	119 (6)	N5—C13—H13A	109.1
C13—N5—C12	112.7 (3)	C14—C13—H13A	109.1
C13—N5—Pb	109.0 (3)	N5—C13—H13B	109.1
C12—N5—Pb	112.3 (3)	C14—C13—H13B	109.1
C13—N5—H5N	105 (4)	H13A—C13—H13B	107.8
C12—N5—H5N	111 (4)	O4—C14—O3	124.8 (5)
Pb—N5—H5N	106 (4)	O4—C14—C13	117.8 (4)
N1—C1—N2	124.7 (4)	O3—C14—C13	117.4 (4)

Symmetry codes: (i) −*x*+1, −*y*+1, −*z*+1.

### Hydrogen-bond geometry (Å, °)

**Table:**

*D*—H···*A*	*D*—H	H···*A*	*D*···*A*	*D*—H···*A*
N2—H2A···O3	0.86 (6)	2.05 (5)	2.880 (6)	162 (5)
N2—H2B···O1W	0.86 (3)	2.16 (4)	2.959 (6)	153 (6)
N4—H4A···O1	0.86 (5)	2.15 (5)	2.946 (5)	155 (5)
N4—H4B···O4^ii^	0.86 (3)	2.07 (5)	2.885 (7)	159 (6)
N5—H5N···O2W	0.87 (5)	2.01 (6)	2.809 (7)	153 (4)
O1W—H11···O2^iii^	0.82 (2)	1.97 (2)	2.783 (6)	171 (5)
O1W—H12···O3^iv^	0.82 (5)	2.11 (4)	2.819 (6)	144 (5)
O2W—H21···O1W^v^	0.82 (7)	2.10 (7)	2.892 (7)	161 (6)
O2W—H22···O4^vi^	0.82 (5)	1.95 (5)	2.766 (6)	168 (7)
C5—H5···O2^vii^	0.93	2.56	3.476 (6)	167

Symmetry codes: (ii) *x*, *y*, *z*+1; (iii) *x*, *y*+1, *z*−1; (iv) −*x*+1, −*y*+2, −*z*; (v) −*x*+2, −*y*+2, −*z*; (vi) −*x*+2, −*y*+1, −*z*; (vii) *x*, *y*+1, *z*.
